# Current opinion on the role of testosterone in the development of prostate cancer: a dynamic model

**DOI:** 10.1186/s12885-015-1833-5

**Published:** 2015-10-26

**Authors:** Xiaohui Xu, Xinguang Chen, Hui Hu, Amy B. Dailey, Brandie D. Taylor

**Affiliations:** 1 Department of Epidemiology & Biostatistics, School of Public Health, Texas A&M Health Science Center, 205A SRPH Administration Building | MS 1266, 212 Adriance Lab Road, College Station, TX 77843-1266 USA; 2 Department of Epidemiology, College of Public Health and Health Professions and College of Medicine, University of Florida, Gainesville, FL USA; 3 Health Sciences Department, Gettysburg College, Gettysburg, PA USA

**Keywords:** Prostate Cancer, Testosterone, Androgen, Dynamic model

## Abstract

**Background:**

Since the landmark study conducted by Huggins and Hodges in 1941, a failure to distinguish between the role of testosterone in prostate cancer development and progression has led to the prevailing opinion that high levels of testosterone increase the risk of prostate cancer. To date, this claim remains unproven.

**Presentation of the hypothesis:**

We present a novel dynamic mode of the relationship between testosterone and prostate cancer by hypothesizing that the magnitude of age-related declines in testosterone, rather than a static level of testosterone measured at a single point, may trigger and promote the development of prostate cancer.

**Testing the hypothesis:**

Although not easily testable currently, prospective cohort studies with population-representative samples and repeated measurements of testosterone or retrospective cohorts with stored blood samples from different ages are warranted in future to test the hypothesis.

**Implications of the hypothesis:**

Our dynamic model can satisfactorily explain the observed age patterns of prostate cancer incidence, the apparent conflicts in epidemiological findings on testosterone and risk of prostate cancer, racial disparities in prostate cancer incidence, risk factors associated with prostate cancer, and the role of testosterone in prostate cancer progression. Our dynamic model may also have implications for testosterone replacement therapy.

## Background

Prostate cancer (PCa) is the most common cancer and the second leading cause of cancer mortality among American men. In 2014, approximately 233,000 men were diagnosed with PCa and 29,480 PCa-related deaths were reported [1]. Despite high incidence and mortality rates of PCa, the biological mechanism related to the development and progression of PCa remains largely unknown. The prostate is an androgen-regulated organ and there is a long-standing interest in understanding the role of androgens in the development of PCa [2, 3]. Androgens are a class of sex steroid hormones which in males, stimulate and control the development and maintenance of male characteristics including growth and function of the prostate. Testosterone and its derivative, dihydrotestosterone (DHT), are the two most abundant androgens in males. Approximately 90 % of testosterone is produced by Leydig cells in the testes and an additional 10 % is produced by adrenal glands [4]. DHT is the primary effector androgen and is converted from testosterone by 5α-Reductase [4]. DHT becomes biologically active by forming the androgen-receptor complex, which is then translocated from the cytoplasm into the cell nucleus to modulate gene expression [5].

The landmark study by Huggins and Hodges in 1941 suggested a direct correlation between circulating levels of testosterone and PCa progression [6]. It was the first study to show that both progression and regression of PCa are testosterone-dependent. These findings led to the prevailing hypothesis that elevated androgen levels increase the risk of PCa. However, Huggins and Hodge’s study only provided evidence on the role of testosterone in the progression of PCa. Therefore, this widely accepted opinion fails to distinguish the role of testosterone in PCa development. Despite more than 70 years passing since the study was conducted, little progress has been made in understanding the role of testosterone in the development of PCa. Furthermore, evidence from epidemiological studies remains controversial. Some studies supported the prevailing opinion that high testosterone levels are associated with an increased risk of PCa [7–11] while others have found negative associations between testosterone and risk of PCa [12–15] or no association [16–24]. A pooled analysis of 19 published studies by Roddam et al. (2008) found no statistically significant association between testosterone and the risk of PCa [25].

All of these studies were guided by a static paradigm, which investigated the relationship between testosterone and PCa at a single point in cases and controls. Although this type of study design is often more feasible, it is not able to examine the relationship between the change of testosterone with age and PCa risk. Furthermore, these studies did not analyze the role of testosterone in the development of PCa in the context of individual variation of testosterone level and are insufficient to examine the complex etiological role of testosterone in the carcinogenesis process of PCa. New paradigms are needed to further understand the current data and to guide us to advance PCa research in the future.

Based on evidence from published studies, we propose a dynamic model as a theoretical framework to understand the relationship between testosterone and the development of PCa. As an illustration, we propose a dynamic model to interpret and improve our understanding of the following: the observed age patterns of PCa, the inconsistent findings from published studies, the racial disparities in PCa incidence, the risk and protective factors for PCa, the role of testosterone in PCa growth and the use of androgen replacement therapy as primary prevention of PCa.

## Presentation of the hypothesis

**Fig. 1:**
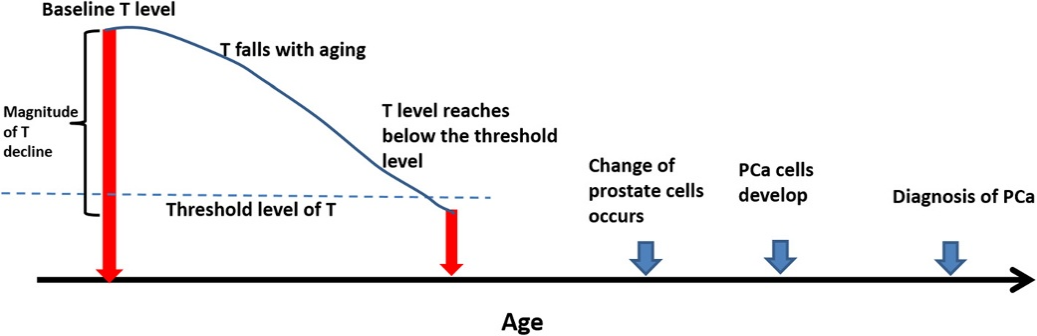
A hypothetical model illustrates the role of age-related declines in testosterone (T) in thedevelopment of PCa

Two key components are included in our dynamic model: the magnitude of the age-related declines in testosterone and the individual-based threshold level of testosterone to maintain the normal function of prostate gland. Our model emphasizes that the absolute value of testosterone measured at a single point is not indicative of PCa risk. Instead, the magnitude of the age-related declines in testosterone is a key factor. The risk of PCa increases when testosterone levels fall below a threshold. As testosterone level falls below the threshold, prostatic cells reach the limit of their compensatory capabilities, thus impairing adaption to lower levels of testosterone and finally triggering the prostatic carcinogenesis process (See Fig. 1).

## Testing the hypothesis

The hypothesis can be tested with prospective cohort studies with longitudinal monitoring testosterone levels in males or retrospective cohort studies with testosterone data available at different ages or stored blood samples collected at different ages available for testing testosterone. With the study design and data, we are able to make a comparison of the patterns of testosterone change over time between cases and controls. For the threshold level, the absolute or relative differences of testosterone levels between young adulthood and at the time of prostate cancer diagnosis may provide important insights about it. In addition, the relative differences of testosterone levels between young adulthood and old age (e.g. 65 years old) may also provide some clues about the threshold level as most of prostate cancer occurs in old age.

## Implications of the hypothesis

### The dynamic model and the effect of age on PCa

After the age of 50, the incidence of PCa increases exponentially with age [26, 27]. Prior studies have yielded rich data regarding the age patterns of testosterone. Testosterone levels are increased in pubertal adolescence, then peak between 30-40 years, and subsequently falling thereafter at the rate of 2–3 % per year [28–30]. In some men, normal prostate cells develop into tumor cells with age after testosterone level reaches blow a certain level (i.e. individual-based threshold), leading to PCa. Figure 1 illustrates the parallels of PCa development and declining testosterone levels. Studies of age patterns of testosterone levels suggest that only a small proportion of individuals have testosterone levels below the threshold before age 50. Consequently, PCa risk is very low among this young population. However, after age 50, the proportion of individuals with testosterone levels below the threshold increases dramatically with each. As a result, PCa risk also increases exponentially.

### The dynamic model and the role of testosterone in the development of PCa

Previous research studies have been limited by static models examining the relationship between testosterone levels and PCa. Most published epidemiological studies measured testosterone at a single point in time, which may contribute to the inconsistent findings in the field.

**Fig. 2:**
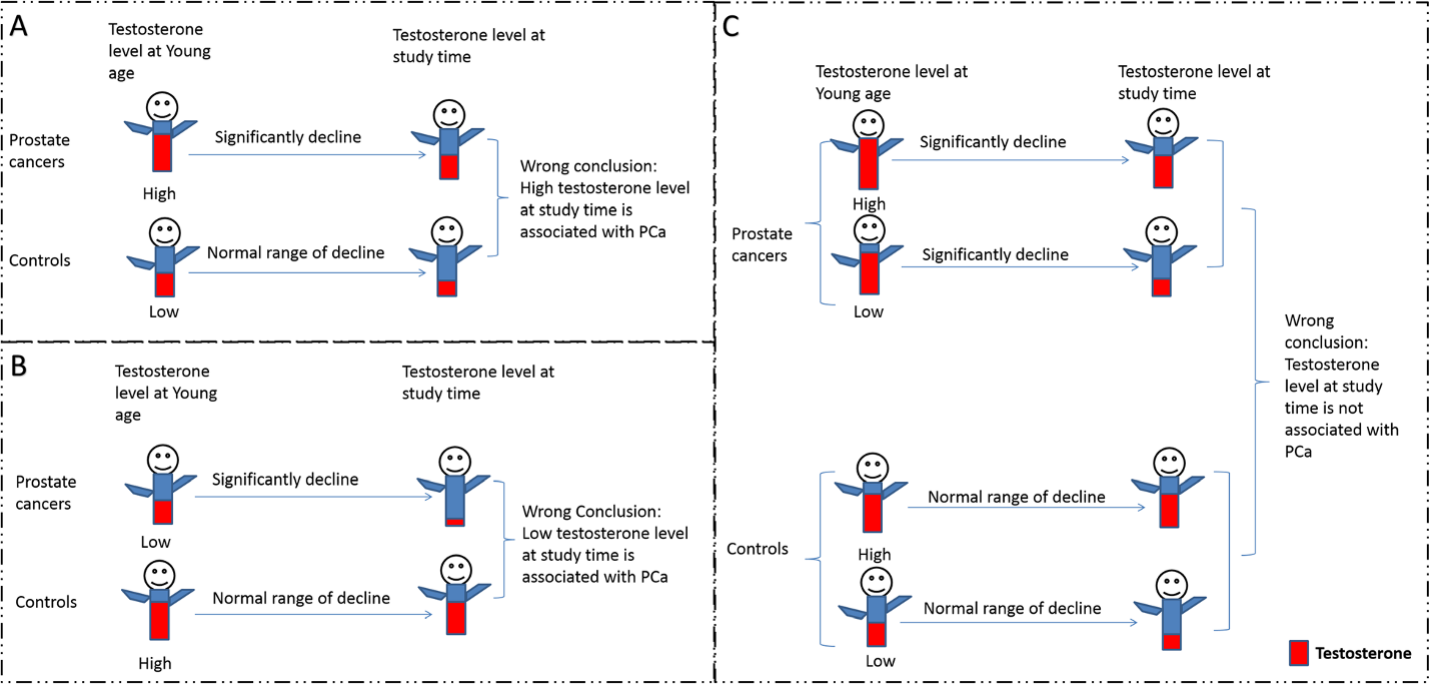
Illustration of the change of testosterone throughout life rather than its level at older age which is implicated in the development of prostate cancer (PCa). **a**. Illustration of a postive association between testosterone and PCa; **b**. Illustration of a negativeassociation between testoterone and PCa; **c**. Illustration of no association between testoterone and PCa

Our dynamic model may help explain conflicting findings. For example, in a group of individuals with PCa who had higher levels of testosterone than others when they were young, their testosterone levels are relatively higher at the time of cancer diagnosis, although they may already have experienced significant declines in testosterone. If such patients are included in research, high testosterone level will be detected as a risk factor for PCa when compared with controls who have relatively lower peak testosterone at young age (See Fig. 2-Scenario A). In contrast, if a group of individuals with PCa had lower peak testosterone when they were young, their testosterone level will further decrease by the time of PCa diagnosis. In this scenario, it is not surprising to observe a negative association between testosterone levels and PCa if these patients are compared with the controls whose dynamic change in testosterone levels follow the pattern for most people in a population (See Fig. 2- Scenario B). If all individuals from Scenario A and Scenario B were analyzed together [25], no association between testosterone levels and PCa is possible (See Fig. 2- Scenario C).

### The dynamic model and PCa racial disparities

Racial disparities in PCa are well documented [31–33]. In the U.S., black males are approximately twice as likely to be diagnosed with PCa compared to white males [34, 35]. However, the determinants of racial disparities in PCa remain unclear. Studies controlling for social impacts of PCa have attempted to link testosterone levels to the racial differences observed in PCa development [36–45], but findings from these studies are inconsistent [36–46]. With the dynamic model, the increased risk of PCa for blacks could be due to more significant reductions in testosterone levels, relative to that of whites. Evidence from previous studies indicates that testosterone levels in black males declines quicker with age when compared to white men. During young adulthood, testosterone levels are higher in blacks than in whites; but the difference diminishes with age and completely disappears after the age of 60 years of age [42, 47, 48]. Thus, the difference in the magnitude between young and older ages may explain, in part, racial differences in PCa risk.

### The dynamic model and risk factors for PCa

According to our dynamic model, any factor that affects testosterone levels with age may play an etiological role in the development of PCa. We applied the dynamic model to explain the observed association between selected known risk factors (physical activity, obesity, zinc levels, and vitamin D levels) and PCa by focusing on the ability of risk factors to mediate changes in testosterone levels. This may occur either by slowing down or accelerating the process of the age-related declines in testosterone.

#### Physical activity and risk of PCa

Studies have shown that both occupational and leisurely physical activity can reduce the risk of PCa [49–53]. Many studies have also found that physical activity can increase testosterone levels, particularly among older men [54–57], contradicting the current paradigm [58]. Our dynamic model more fully allows for the idea that physical activity prevents PCa by increasing testosterone levels, slowing down the age-related declines in testosterone.

#### Obesity and risk of PCa

Evidence from a meta-analysis and systematic reviews suggest that obesity is linked with an increased risk of PCa [59–61], yet explanations for this relationship are weak. Based on our dynamic model, we have at least two possible explanations for this relationship: (1) Being overweight/obese accelerates the age-related declines in testosterone, or (2) overweight/obesity is simply an indicator of accelerated testosterone declines [62]. Findings from epidemiological studies indicate that compared to men with normal weight, obese men have lower testosterone [63, 64]. However, the underlying mechanisms are complex and include many factors such as inactive lifestyle, diet and accelerated testosterone metabolism. For example, studies found that adipose tissue has a strong ability to convert androgen into estrogen [65]. Moreover, the increased androgen-estrogen conversion suppresses the release of luteinizing hormone, reducing the production of testosterone by Leydig cells through a negative hypothalamic-pituitary-gonadal axis feedback loop [66, 67]. Thus, accelerating testosterone metabolism through fat tissues could be one mechanism explaining the mediating role of testosterone in the associations between overweight/obesity and PCa. It is also possible that testosterone levels at baseline are associated with the development of obesity; thus, a detailed time course evaluation of testosterone may be required to fully understand the relationship.

#### Zinc and risk of PCa

Zinc is the most abundant trace mineral in the body [68, 69], playing a pivotal role in immune function, antioxidant activities, hormonal function and cellular activities [70–72]. The prostate has the highest concentration of zinc in the male body secreting large amounts of the mineral into prostatic fluid [73]. Thus, there is a growing interest in investigating the role of zinc in the carcinogenesis and pathogenesis of PCa [74]. Many epidemiological studies have reported marked decreases of zinc levels in PCa tissues versus normal prostate tissues [75–83]. Furthermore, studies suggest that high zinc levels are associated with antitumor effects [84, 85]. Studies have shown that zinc is important for testosterone production and zinc supplementation can dramatically raise systemic testosterone levels [86–90]. While many biological pathways may be involved in the protective effects of zinc against PCa, the inverse association between zinc and PCa is consistent with the dynamic model we proposed. According to our model, the protective effect of zinc on PCa could be through its role in slowing down the age-related declines in testosterone.

#### Vitamin D deficiency and risk of PCa

Research indicates that exposure to UV radiation is inversely correlated with PCa incidence and mortality [91–93] and that vitamin D protects against prostate cancer [94–98]. Although the underlying biological mechanisms between vitamin D and PCa may be complex, our dynamic model provides an explanation. Vitamin D may reduces PCa risk by slowing down the age-related declines in testosterone. Studies have shown that vitamin D can increase testosterone levels in males [99–102]. In addition, vitamin D deficiency is more prevalent among blacks than other racial groups [103, 104], which may help explain more rapid testosterone declines among blacks, and may also contribute to racial disparities in PCa risk.

In summary, all the factors that are reported to be associated with PCa, as described above, are involved directly or indirectly with levels of testosterone and changes with age. The dynamic model, which proposes that the magnitude of age-related declines in testosterone plays an essential role in the genesis of PCa, may help explain the observed associations between these factors and risk of PCa. As the dynamic model suggests, a risk factor may be in the causal pathway of PCa development through acceleration of age-related declines in testosterone, while protective factors may slow down the process. Observed relationships between the risk/protective factors discussed above and testosterone are consistent with the dynamic model.

### The dynamic model and the role of testosterone in PCa growth

#### Different roles of testosterone in the onset and progression of PCa

To date, no documented epidemiological studies have distinguished testosterone as a cause of PCa from a promotor of PCa growth. One advantage of our dynamic model is that it can be used to assess the role of testosterone in the onset of PCa. As the model suggests, the prostatic carcinogenesis may be a process by which the normal prostate cells first adjust themselves to progressive declining testosterone levels at the cellular and receptor levels. As testosterone levels fall below the threshold when normal prostate cells are not able to make additional adjustments without mutations, some of the normal prostate cells may evolve into cancer cells. If additional testosterone is added before reaching the threshold level, it may change the course of the disease. Among the mutated cancer cells, some of them may become testosterone sensitive and increases in testosterone may therefore promote these cancer cells to grow. This notion is supported by evidence that castration (removal of endogenous testosterone) can inhibit PCa progression [6, 105], while administration of exogenous testosterone can promote PCa progression [106, 107]. Therefore, our dynamic model can also be used to interpret the seemingly conflicted findings that higher testosterone can prevent PCa onset but promote PCa progression after the disease occurs.

#### The dynamic model and androgen signaling pathway

Androgen receptor (AR) signaling plays an important role in the normal development and homeostasis of the prostate gland [108, 109]. AR is a nuclear receptor that binds testosterone. The androgen-AR is directly involved in a number of cellular processes that may lead to PCa genesis, including the regulation of cell cycle, adhesion, apoptosis and extracellular matrix remodeling and metabolism [110]. According to our dynamic model, when testosterone levels reach the threshold, all biochemical processes that are involved with androgen-AR may be altered. Moreover, the testosterone threshold for PCa of an individual may also be determined by the total number and characteristics of AR in normal prostate cells during young adulthood. The hypothesized threshold could be higher for individuals with higher testosterone than those with lower testosterone during young adulthood. Evidence from reported studies tends to support this hypothesis. For example, evidence from randomized controlled trials indicates that most prostate cancers that initially responded to androgen deprivation therapy develop into androgen-independent cancer after a few years of treatment [111–114]. The mechanisms by which tumor cells escape androgen ablation and become independent of the need for androgen might not be to the same as that of normal prostate cells turning into cancer cells. However, they indicate that changes in testosterone may lead to changes at the cellular and molecular levels. Further investigation is needed to confirm these hypotheses by mimicking testosterone decline with aging *in vivo* or *in vitro* and studying its effects on changes in prostate cells.

### The dynamic model and testosterone replacement therapy

The question whether testosterone replacement therapy is a risk factor for PCa remains controversial [115]. If confirmed, our dynamic model suggests that testosterone replacement therapy should be provided before testosterone levels drop below the threshold.

Some potential and practice-related questions that also remain include dosage and timing for testosterone replacement therapy to prevent PCa. According to our dynamic model, the purpose of testosterone replacement therapy is to compensate the age-related declines in testosterone and to maintain testosterone levels above the threshold. In our dynamic model, the concept of individual-based hypothesized thresholds of testosterone provides a conceptual framework supporting further research to determine the protocol for individualized PCa prevention using exogenous testosterone. Individual variation is important to understand, as peak testosterone levels may influence threshold levels, and some individuals may have stronger compensatory function.

The timing for testosterone replacement therapy is also important to consider. For primary prevention of PCa, testosterone replacement therapy needs to begin prior to the onset of PCa, when testosterone levels are still above the threshold. If some prostate cells have already become cancer cells, administration of testosterone may promote PCa growth. Given the challenges with determining individual thresholds, longitudinal monitoring of testosterone levels may be another approach to determining the appropriate dosage and timing of testosterone replacement therapy. Examination of testosterone levels in the general population may need to start before age 30 since the incidence of PCa in autopsy studies has been reported to be as high as 17 % in individuals less than 30 years old [116]. If possible, examination of testosterone levels in prostate tissue may be more informative. When testosterone level falls below a certain percent of the peak level of testosterone, testosterone replacement therapy can restore testosterone levels. However, a recent clinical trial found that intraprostatic testosterone and dihydrotestosterone levels did not significantly increase after administration of supraphysiologic doses of testosterone in patients with symptomatic hypogonadism during the 6-months of follow-up [117]. This finding suggests that the circulating levels of testosterone may be less affected than testosterone levels in the prostate. Nonetheless, the intraprostatic levels of testosterone and dihydrotestosterone declined in the control group, suggesting that treatment is working to increase the T and DHT levels. Without treatment, those with symptomatic hypogonadism may not have stable or slightly higher levels of testosterone and dihydrotestosterone. Testosterone replacement therapy has often been applied to treat male hypogonadism. Studies indicate that long-term testosterone replacement appears to be a safe and effective for male hypogonadism [118–121]. Receiving long-term testosterone replacement therapy for hypogonadism men is not associated with an increased risk of PCa [122–124]. In addition, studies also found that men with benign prostate biopsies do not have increased in prostate specific antigen or a significantly increased risk of cancer compared to normal men after one year of testosterone replacement therapy [125]. All findings suggest that testosterone replacement therapy may not be harmful to prostate health. Of course, it remains to be proven that testosterone has any role in prostate carcinogenesis outside of causing growth of pre-existing PCa.

### Summary

PCa is a killer of millions of men in the United States and across the globe. The dynamic model provides a novel conceptual framework to explain contradictory findings from reported epidemiological studies. Our dynamic model suggests that a significant decline in testosterone levels with age may indicate the role of testosterone in the development of PCa. Our theory suggests a new direction for epidemiological studies to examine the relationship between testosterone levels and risk of PCa by targeting the magnitude of age-related declines in testosterone rather than testosterone levels measured at a single point in time. Some fundamental changes in study design are required. If the model is confirmed, it will provide important insights in the etiology and primary prevention of PCa.

## Abbreviations

AR: Androgen receptor
DHT: Dihydrotestosterone
PCa: Prostate cancer
UV: Ultraviolet radiation
